# *Lactobacillus* for the treatment and prevention of atopic dermatitis: Clinical and experimental evidence

**DOI:** 10.3389/fcimb.2023.1137275

**Published:** 2023-02-16

**Authors:** Anni Xie, Ailing Chen, Yuqing Chen, Zichen Luo, Shanyu Jiang, Daozhen Chen, Renqiang Yu

**Affiliations:** ^1^ Department of Neonatology, Wuxi Maternity and Child Health Care Hospital, Wuxi School of Medicine, Jiangnan University, Wuxi, China; ^2^ Research Institute for Reproductive Health and Genetic Diseases, Wuxi Maternity and Child Health Care Hospital, Wuxi School of Medicine, Jiangnan University, Wuxi, China; ^3^ Department of Child Health Care, Wuxi Maternity and Child Health Care Hospital, Wuxi School of Medicine, Jiangnan University, Wuxi, China

**Keywords:** atopic dermatitis, *Lactobacillus*, type 2 helper cells, gut microbiota, immunomodulation

## Abstract

Atopic dermatitis (AD) is a chronic inflammatory skin disease, accompanied by itching and swelling. The main pathological mechanism of AD is related to the imbalance between Type 2 helper cells (Th2 cells) and Type 1 helper cells (Th1 cells). Currently, no safe and effective means to treat and prevent AD are available; moreover, some treatments have side effects. Probiotics, such as some strains of *Lactobacillus*, can address these concerns *via* various pathways: i) facilitating high patient compliance; ii) regulating Th1/Th2 balance, increasing IL-10 secretion, and reducing inflammatory cytokines; iii) accelerating the maturation of the immune system, maintaining intestinal homeostasis, and improving gut microbiota; and iv) improving the symptoms of AD. This review describes the treatment and prevention of AD using 13 species of *Lactobacillus*. AD is commonly observed in children. Therefore, the review includes a higher proportion of studies on AD in children and fewer in adolescents and adults. However, there are also some strains that do not improve the symptoms of AD and even worsen allergies in children. In addition, a subset of the genus *Lactobacillus* that can prevent and relieve AD has been identified *in vitro*. Therefore, future studies should include more *in vivo* studies and randomized controlled clinical trials. Given the advantages and disadvantages mentioned above, further research in this area is urgently required.

## 1 Introduction

**Figure 1:**
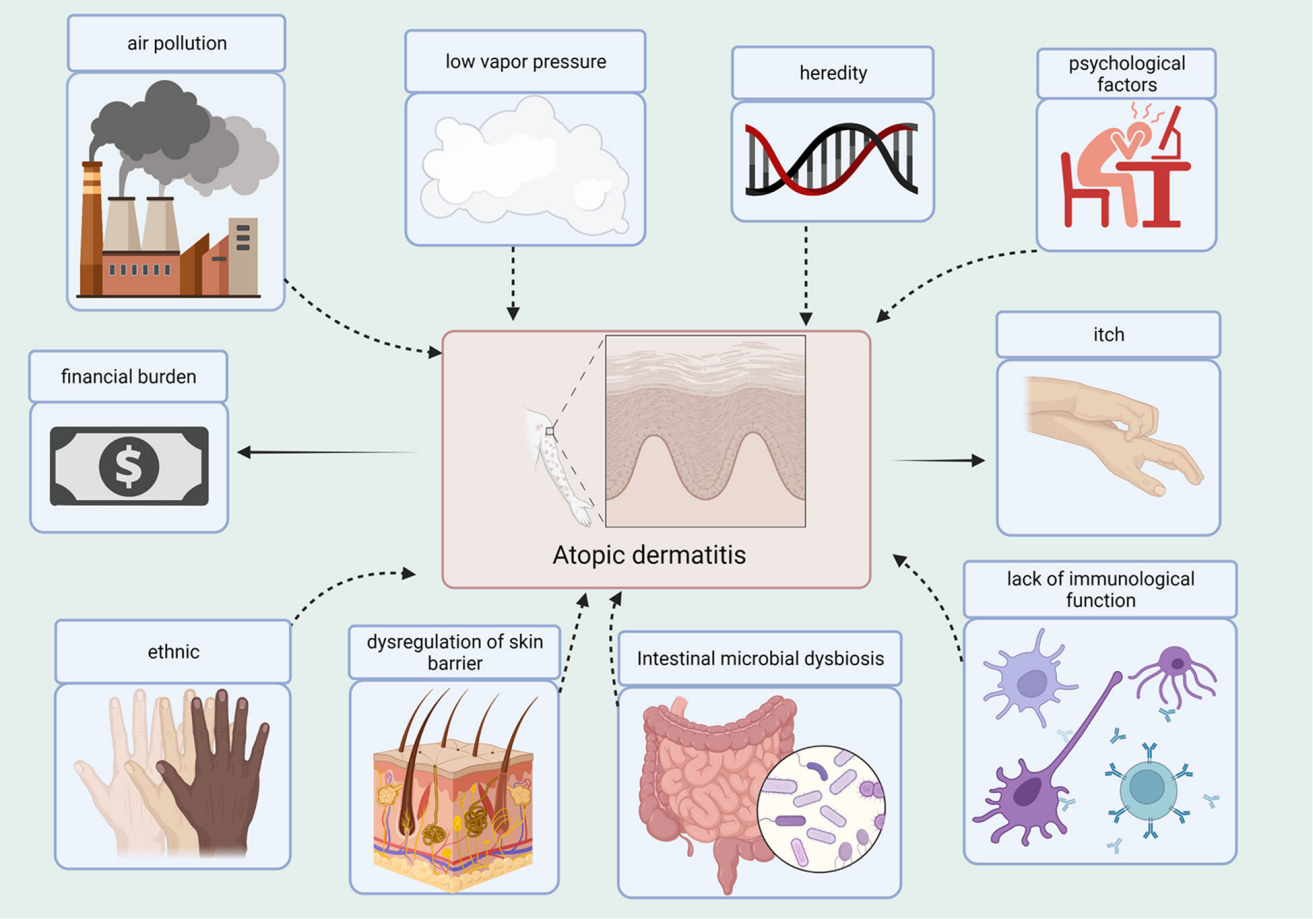
Causes and Consequences of the AD. Several factors contribute to the development of AD, such as air pollution, low vapor pressure, heredity, psychological factors, ethnicity, dysregulation of the skin barrier, dysbiosis of gut microbes, and lack of immune function. AD contributes to the itchiness and financial burden.

Atopic dermatitis (AD) is a chronic inflammatory skin disease, and patients frequently experience complications from concurrent allergic conditions. The annual incidence of AD has increased in the recent years, particularly in children. Based on the Finnish national database, the prevalence of AD varies by age group, with the highest prevalence in the age group 0-14 years (47.46%), followed by that in 15-60 year olds (43.74%) (Salava et al., 2022). The actual prevalence of AD between 6 months and 12 years has been reported at 11% in Israel (Weil et al., 2022). One report showed that the incidence of AD in infants aged 3 months in China was 40.81% (Guo et al., 2019). Patient quality of life can be severely affected, as AD causes scratching, itching, and a rash. The financial burden on households increases with disease severity (Weil et al., 2022). The incidence of AD is strongly correlated with heredity and environment (**Figure 1**). In other words, people with AD often have a family history of AD, and the incidence of AD may increase when the father or mother has a particular allergy. Although the exact mechanisms of AD have not yet been elucidated, impaired immunological function and dysregulation of the skin barrier are considered to be the primary pathogenic mechanisms (Yang et al., 2020). Concurrently, environmental factors such as poor eating habits, sudden lifestyle changes, and certain allergenic stimuli are also associated with AD. A climatic conditions closely associated with an elevated risk of AD is low vapor pressure (Yokomichi et al., 2022). In Chongqing, China, infants exposed to polluted air are at an increased risk of developing AD (Luo et al., 2022). Psychological factors also play an important role in AD development; subsequently, AD leads to fluctuating depressive symptoms (Chatrath et al., 2022). As a result, individuals with AD can be caught in a vicious cycle.

Although the pathogenesis of AD is not clear, decades of research indicate that the mechanisms of AD can potentially be attributed to genetic factors, environmental exposures, impaired skin barrier, abnormal immune function, and microbial imbalances. This suggests that AD is a systemic organ-related allergic disease. One study demonstrated an increased probability of early AD in maternal offspring due to dysregulated interferon signaling cascade (Schedel et al., 2023). Patients with high-risk genes tend to develop AD earlier (Hikino et al., 2022). A notable manifestation of AD is pruritus, which causes patients to scratch vigorously (Yosipovitch et al., 2020; Steinhoff et al., 2022). After skin rupture, the epidermal barrier is damaged, and antigens penetrate the skin, producing chemokines and inflammatory mediators (IL-25, IL-33, and thymic stromal lymphopoietin). Th cells polarize to Th2 and produce IL-4, IL-13, and IL-5 (Cosmi et al., 2019; Renert-Yuval and Guttman-Yassky, 2020). Th1, Th17, Th22, and other pathways are activated; a variety of cytokines and growth factors involved in inflammatory immune responses through the Janus kinase (JAK) pathway are produced and enhance Th2 cell differentiation (Bao et al., 2013; Rerknimitr et al., 2017). Scratching coupled with endogenous and other exogenous triggers, such as histamine, proteases, substance P, various interleukins, and environmental allergens leads to keratinocyte activation, and intensified skin inflammation. Inflammatory mediators and multi-pathway inflammation cause intensive scratching and further damage to the skin barrier. Moreover, bacteria take advantage of the situation and the vicious circle continues. Under physiological conditions, the skin microenvironment maintains immune homeostasis and reduces skin colonization by pathogenic bacteria. Diversity of the gut microbiome is significantly lower in infants with AD (Abrahamsson et al., 2012; Penders et al., 2013). The presence of *Staphylococcus aureus* was detected on the skin of 90% of patients with AD, and this pathogen can lead to disease progression (Powers et al., 2015; Blicharz et al., 2019). *Staphylococcus aureus* secretes staphylococcal enterotoxins A, B, and C and toxic shock syndrome toxin 1 (TSST-1), which activate lymphocytes and macrophages (Nakatsuji and Gallo, 2019). In addition, staphylococcal enterotoxin B promotes the expression of IL-31. IL-31 inhibits the expression of polyfilament proteins and antimicrobial peptides, which favor *Staphylococcus aureus*. Importantly, IL-31 is a key factor in itching (Meng et al., 2018). Previous studies showed that mediators produced by *Staphylococcus aureus* promotes adhesion, colonization, and spread to the skin. These mechanisms are complex and interact. In future, scientists may also identify new mechanisms that are yet to be discovered.

The gut plays an important role in the immune response. At the same time, Natural killer (NK) T cells, innate lymphoid cells, and intestinal flora regulate each other to maintain intestinal homeostasis and normal immune function (Cairo and Webb, 2022). In addition, healthy gut flora has a protective effect against food allergies (Méndez et al., 2021). Disturbances of the intestinal microbiome in early infancy worsen immune dysfunction in children with AD (Lee et al., 2022). In addition, a study showed that transplanting fecal microbiota to restore gut ecology provides a new method for treating AD (Kim et al., 2021). Lower microbial diversity has been associated with a higher incidence of AD (Galazzo et al., 2020). Moreover, greater severity of clinical manifestations in patients with AD has been associated with a lower number of *Bifidobacteria* in the intestine (Watanabe et al., 2003). Conversely, higher amounts of pathogenic *Clostridium difficile* have been detected in the stool of patients with AD (Penders et al., 2007). In one region of Brazil, children with AD have a higher prevalence of *Clostridium difficile* and a lower abundance of *Lactobacillus* (Melli et al., 2020). *Clostridium difficile* causes a decrease in beneficial bacteria, loss of immune function, and increased intestinal permeability. Studies have shown that colonization of the gut flora precedes AD changes (Galazzo et al., 2020); therefore, a timely intervention in the gut microbiota could be a promising preventive approach. Diet has an important impact on the colonization of gut microbes in early infancy (Galazzo et al., 2020). In early infancy, microbes are primarily affected by type of delivery; however, food becomes an important factor starting at 13 weeks (Galazzo et al., 2020). Food allergies and AD are closely related, and approximately one-third of children have both AD and food allergies (Hui-Beckman et al., 2023). Food-induced AD most likely occurs in children with severe AD (Robison and Singh, 2019). Food allergies increase the permeability of the intestine, making it easier for allergens to trigger the submucosal immune system through the intestinal barrier (Lee et al., 2013; Gao et al., 2021). Cytokines and inflammatory mediators produced after the activation of the immune system further increase intestinal permeability. The interaction between the gut microbiota and skin has been called the gut-skin axis by some authors (Mahmud et al., 2022; Varela-Trinidad et al., 2022). Healthy gut flora is beneficial for healthy skin.

Various treatments have been used in AD, including topical glucocorticoids and immunosuppressive agents, phototherapy, and narrow-spectrum ultraviolet radiation B. AD is a chronic inflammatory disease that affects patients with impaired skin barrier function. The long-term administration of topical corticosteroids carries a high risk. Topical corticosteroids are the mainstream treatment for moderate to severe AD; however, they have side effects, such as hormonal dermatitis, when used in large, long-term doses in combination with potent hormonal creams. In addition, prolonged use of topical corticosteroids may cause serious side effects, such as adrenal insufficiency (Böckle et al., 2014). Additionally, patients hesitate to use glucocorticoids (Maghen et al., 2019). Immunosuppressant treatment for AD may cause conjunctivitis (Wollenberg et al., 2018) or lymphopenia (Bakker et al., 2018) in some patients. Narrow-spectrum ultraviolet radiation B has a relieving effect on AD (Ben Mordehai et al., 2022); however, long-term use may cause abnormal skin reactions. Moreover, some biological drugs are expensive and have side effects (Puar et al., 2021). Patients receiving dupilumab treatment were reported to suffer from eye discomfort (Miniotti et al., 2022). Owing to the above reasons, safe and effective treatments for AD remain limited. Recently, treatment with probiotics has been proposed to regulate the gut microbiome in AD. The gut microbiome plays an essential role in the maintenance of host homeostasis and immunomodulation. Imbalances in microbial flora can contribute to many diseases. Intestinal microbial dysbiosis is the leading cause of AD-like symptoms (Kim et al., 2020a). Oral administration of *L. sakei* proBio65 (Rather et al., 2021) and *L. salivarius* LS01 can improve the quality of life of children (Niccoli et al., 2014) and adults (Drago et al., 2011) with AD. In an experimental model, maternal mice and their offspring orally supplemented with *L. reuteri* Fn041 maintained the balance of the immune response to prevent AD (Qi et al., 2022; Zhao et al., 2022; Zhou et al., 2022).

*Lactobacillus* is a naturally occurring rod-shaped bacterium that is a part of the normal flora in some organs of humans, animals, and plants. The storage conditions for *Lactobacillus* are simple. *L. sakei* proBio65 live and inactivated bacteria can improve AD symptoms and enhance the function of skin barrier (Jeong et al., 2020a; Rather et al., 2021). Administration *L. paracasei* KBL382 alleviated AD by modulating immune responses (Kim et al., 2020a). *L. paracasei* KBL382 reduced serum levels of immunoglobulin E (IgE) and immune cell infiltration (Kim et al., 2020a). Moreover, supplementation with *L. rhamnosus* HN001 substantially reduced the cumulative prevalence of AD (Wickens et al., 2008). These findings suggest that *Lactobacillus* may provide an alternative strategy for the treatment and prevention of AD. In this review, we focus on the role of *Lactobacillus* as a novel therapeutic agent for AD.

## 2 Classification of *Lactobacillus* and its mechanism of prevention and treatment of AD

**Figure 2:**
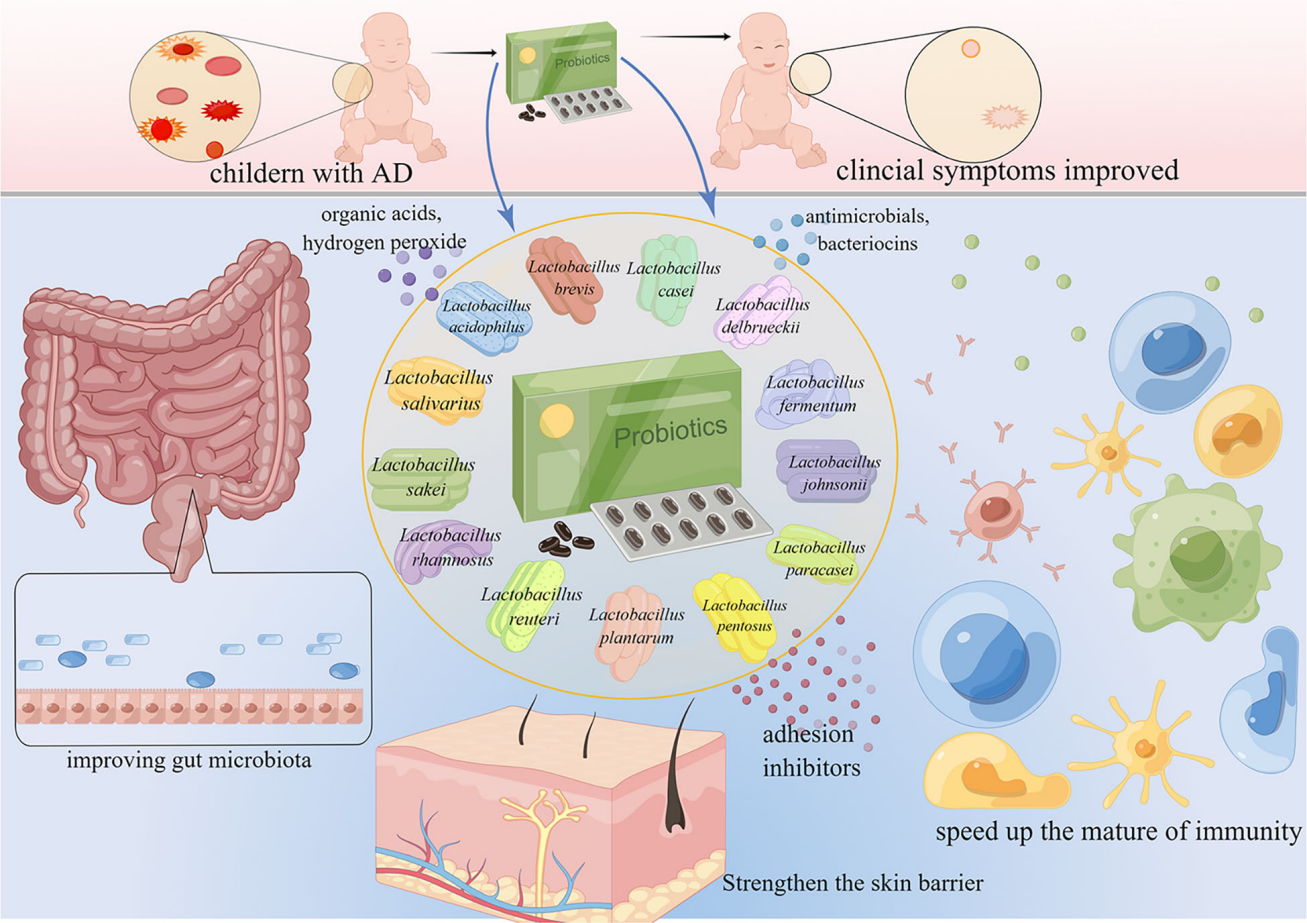
*Lactobacillus* for the treatment and prevention of AD. AD is common in children. *Lactobacillus* accelerates the maturation of the immune system, maintains intestinal homeostasis, improves the gut microbiome, and ultimately improves the symptoms of AD.

Probiotics are living microorganisms such as *Lactobacillus* spp., *Bifidobacterium* spp., *Enterococcus* spp., *Streptococcus* spp., *Propionibacterium* spp., *Bacillus cereus* spp., and *Saccharomyces boulardii* that benefit the host. *Lactobacillus* spp. is the most widely used probiotic microorganism. The genus *Lactobacillus* includes more than 200 species (Sun et al., 2015), and can be subdivided into at least 24 phylogenetic groups (Zheng et al., 2015). Several *Lactobacillus* species have been studied for AD prevention and treatment (**Figure 2**), including *L. rhamnosus*, *L. plantarum*, *L. acidophilus*, *L. sakei*, *L. reuteri*, *L. salivarius*, *L. paracasei*, *L. casei*, *L. delbrueckii*, *L. fermentum*, *L. johnsonii*, *L. pentosus*, and *L. brevis*. These *Lactobacillus* species have been reported to produce a variety of substances, such as organic acids, hydrogen peroxide, low-molecular-weight antimicrobials, bacteriocins, and adhesion inhibitors. *Lactobacillus* products stimulate innate immunity and promote balanced microbial communities through the competitive rejection and antimicrobial activity against pathogenic bacteria (Goldstein et al., 2015). Administration of *Lactobacillus* decreased the serum levels of IgE (Sunada et al., 2008; Wakabayashi et al., 2008; Tanaka et al., 2009; Prakoeswa et al., 2017), and achieved a balance of Th1/Th2 (Wickens et al., 2008; Kwon et al., 2018; Kim et al., 2019a; Zhao et al., 2022). Moreover, the intestinal barrier (Wickens et al., 2008; Kim et al., 2020a; Zhao et al., 2022), immune function (Wickens et al., 2008; Kim et al., 2019a; Kim et al., 2020a) and skin barrier (Mariman et al., 2016) have been improved after the administration of *Lactobacillus.* The mechanisms of the 13 kinds of *Lactobacillus* are listed in **Table 1**. *Lactobacillus* shows certain effects on both animals and humans with AD (**Tables 2**, **3**). *Lactobacillus* has high economic value in biotechnology, food production, and therapeutic applications.

**Table 1:** Mechanism of action of *Lactobacillus* in the treatment of AD.

Probiotic	strain	Mechanism of action	Reference
***L. rhamnosus***	RHT3201	decrease of eosinophil cationic protein, eosinophil count, IL-31, and serum IgE concentration	(Lee et al., 2016; Jeong et al., 2020a)
	HN001	enhanced gut barrier function, influence on the immune system and a more balanced Th1/Th2 immune profile	(Wickens et al., 2008)
	LGG	decreased IL-10 protection against pathogenic macromolecules in the gut and accelerated immunological maturation, regulation of the immune response, stimulation of peripheral blood cells to secrete IL-10 and transforming growth factor β	(Kalliomäki et al., 2003; Sawada et al., 2007; Boyle et al., 2008; Marsella, 2009; Nermes et al., 2011; Marsella et al., 2012; Carucci et al., 2022)
	CGMCC 1.3724 (LPR)	reduction of plasma total IgE, upregulation of IFN-γ production at the skin level	(Tanaka et al., 2009)
***L. acidophilus***	L-92	activation of regulatory T cells and Th1 cells, decrease of eosinophil count and increase of change ratio for serum TGF-β, prevention of IgE-mediated hypersensitivity, modulation of Th1/Th2 balance	(Shah et al., 2010; Torii et al., 2011; Inoue et al., 2014; Yamamoto et al., 2016)
	L-55	decrease of serum total IgE level	(Sunada et al., 2008)
	LAVRI-A1	increase of allergen sensitization in infants	(Taylor et al., 2007)
***L. plantarum***	MG4221	inhibition of inflammatory and allergic reactions and regulation of the NF-κB/MAPK pathways in keratinocytes	(Hong et al., 2021a)
	LM1004	decrease of mRNA levels of Th2 and Th17 cell transcription factors, increase of transcription factors of Th1 and Treg cells, galactin-9, filaggrin, enhanced immunomodulation	(Kim et al., 2019a)
	CJLP133	upregulation of total IgE levels, increased TGF-β expression, increased proportion of regulatory T cells at baseline	(Kim et al., 2017)
	IS-10506	decreased levels of serum IgE, IL-4, and IL-17, increased the levels of Foxp3+ to IL-10 ratio, downregulate Th2 adaptive immune response	(Prakoeswa et al., 2017)
	NCIMB8826	amelioration of skin pathology, improvement of skin barrier integrity, skin thickening, and diminished excoriations	(Mariman et al., 2016)
***L. sakei***	ProBio65	increased expression of Foxp3+ transcription factor, Modulation of the expression levels of inflammatory cytokines IL-10 and IL-12, inhibition of mast cell activation, improvement of allergen-induced skin inflammation, downregulation of IgE and IL-4 production	(Park et al., 2008; Kim et al., 2013; Rather et al., 2021)
	KCTC 10755BP	decreased chemokine levels	(Woo et al., 2010)
	WIKIM30	modulation of allergic Th2 responses, enhanced Treg generation and increased relative abundance of intestinal bacteria that are positively related to Treg generation	(Kwon et al., 2018)
***L. reuteri***	Fn041	regulation of systemic Th1 and Th2 cytokine ratios and promotion of CD4+CD25+Foxp3+ regulatory T cell proliferation in mesenteric lymph nodes, regulation of intestinal microbiota	(Zhao et al., 2022)
	JCM 1112	dependent on the presence of Toll-like receptor 2 and the induction of TNF-α-induced protein 3 and cylindromatosis in HaCaT cells	(Kawahara et al., 2018)
	ATCC55730	modulation of the *in vivo* the cytokine pattern at an extra-intestinal site	(Miniello et al., 2010)
***L. salivarius***	LS01	Initiation of intestinal immunity, rebalancing of the changed intestinal microbiota, modulation of Thl/Th2 cytokine profiles	(Drago et al., 2011; Drago et al., 2012; Niccoli et al., 2014)
	PM-A0006	immune-modulating effects	(Wu et al., 2012)
***L. paracasei***	CBAL74	steroid sparing effect	(D'Auria et al., 2021)
	KBL382	increased immunosuppressive response and modified metabolic functions of gut microbiota	(Kim et al., 2020a)
	WK3001	diminished mast cell infiltration and plasma IgE levels, suppression of immediate hypersensitivity reactions and IL-4 mRNA expression in the auricles	(Wakabayashi et al., 2008)
***L. casei***	KCTC 12398BP	isolation of P14 protein decreased the levels of IL-4 in RAW264.7 and Balb/c splenocytes *in vitro* and ex vivo	(Kim et al., 2015b)
	DN-114001	effect on gut microbiota	(Klewicka et al., 2011)
	JCM 1134^T^	modulation of the serum levels of IgE and cytokines and eosinophil count	(Ogawa et al., 2006)
***L. delbrueckii***	subsp. Bulgaricus LB-2	influence on both Th1 and Th2 through induction of Treg cells and secretion of IL-10	(Sheikhi et al., 2017)
	OLL1073R-1	attenuation of IL-6 secretion from lymph node cells and reduced IL-6 levels in inflamed tissues, such as auricles	(Kano et al., 2013)
	R-037	induce IL-12 and Th1	(Watanabe et al., 2009)
***L. fermentum***	VRI 003 PCC™	increased TNF-α responses to both heat-killed *Staphylococcus aureus* and heat-killed *Lactobacillus*	(Prescott et al., 2005)
***L. johnsonii***	NCC533	reduction of the gene expression of proinflammatory cytokines (IL-8, IL-12 and IL-23) and CD86	(Inoue et al., 2007)
***L. brevis***	NS1401	restoration of the Th1/Th2 balance through enhancing Th1-mediated immunity	(Choi et al., 2017)
	SBC8803	increased IgE production, increased production of immunosuppressive cytokines such as IL-10 and TGF-b1	(Segawa et al., 2008a)

**Table 2:** Effects of *Lactobacillus* on the clinical manifestations of AD.

Probiotic	strain	Participants	Interventions	Outcome	Reference
***L. rhamnosus***	RHT3201	100 children (aged 1–12 years) with moderate AD	1.0 × 10^10^ CFU (no details were provided)	decreased SCORAD total score and levels of eosinophil cationic protein	(Jeong et al., 2020a)
	HN001	mothers from 14–16 weeks gestation	6×10^9^ CFU/d, from 14-16 weeks gestation until 6 months post-partum if breastfeeding	no significant differences	(Wickens et al., 2018)
		High risk infants	6×10^9^ CFU/d, 9×10^9^ CFU/d, indirectly from 35 weeks gestation until 6 months after birth if breastfeeding, and directly from birth until 2 years	decreased cumulative prevalence of eczema, SCORAD score, and skin prick tests sensitization	(Wickens et al., 2013)
		425 children	6 ×10^9^ CFU, 9 ×10^9^ CFU/d, from 35 weeks gestation until birth, continued to 6 months after birth in mothers if breastfeeding, and from birth until 2 years in all infants	reduced cumulative prevalence of eczema at 4 years	(Wickens et al., 2012)
		Pregnant women, 474 infants at risk of allergic disease	6×10^9^ CFU/d, pregnant women from 35 weeks gestation until 6 months if breastfeeding, infants receive the same treatment from birth to 2 years	reduced risk of eczema	(Wickens et al., 2008)
	LGG	250 pregnant women carrying infants at high risk of allergic disease	1.8×10^10^ CFU/d, from 36 weeks gestation until delivery	no significant differences	(Boyle et al., 2011)
		100 AD patients (aged 6– 36 months)	1×10^10^ CFU/d, for 12 weeks	reduced SCORAD scores	(Carucci et al., 2022)
		159 mothers, 132 children at high risk	1×10^10^ CFU/d, for 4 weeks	improved AD	(Kalliomäki et al., 2003)
		39 infants with AD	3.4×10^9^ CFU/d	increased proportions of CD19^+^ CD27^+^ B cells and fewer infection	(Nermes et al., 2011)
		131 children (6–24 months old)	10^10^ CFU, 6 months	no significant differences	(Rose et al., 2010)
		35 infants (aged less than 1 year) with atopic eczema	4 weeks	no significant differences	(Salmi et al., 2010)
		11 adults and 73 infants	1.8×10^10^ CFU/d, for 7 days. 1.8×10^10^ CFU/d, from 36 weeks gestation until delivery	no significant differences	(Boyle et al., 2008)
		105 pregnant women	5×10^9^ CFU, twice daily	no significant differences	(Kopp et al., 2008)
		infants (aged 3–12 months) with mild-to-moderate AD	5×10^9^ CFU, for 12 weeks	no significant differences	(Grüber et al., 2007)
		54 infants (aged 1–55 months) with moderate to severe AD	10×10^9^ CFU/d, for 8 weeks	no significant differences	(Fölster-Holst et al., 2006)
***L. acidophilus***	L-92	57patients with AD(≥ 16 years)	20.7 mg/d, for 24 weeks	decreased investigator global assessment, eczema area and severity index, and AD score	(Yamamoto et al., 2016)
		49 AD patients (aged ≥ 16 years)	20.7 mg/d, for 8 weeks	decreased SCORAD scores and eosinophil count	(Inoue et al., 2014)
		20 children (age 4–15 years)	3×10^10^ CFU/d, for 8 consecutive weeks	ameliorated symptoms of AD in Japanese children and effect on serum concentrations of thymus	(Torii et al., 2011)
	LAVRI-A1	153 children	3×10^9^/d, from birth to 6 months.	no significant differences	(Prescott et al., 2008)
		231 pregnant atopic women and babies	3×10^9^/d, for the first 6 months of life	no significant differences	(Taylor et al., 2007)
		178 children	3 × 10^9^ CFU/d, for 6 months	no significant differences	(Jensen et al., 2012)
***L. plantarum***	CJLP133	76 children (median age of 7.1 years) with moderate-to-severe AD	1×10^10^ CFU/d, for 12 weeks	increased proportion of Treg cells with concurrent decrease in TGF-β mRNA expression	(Kim et al., 2017)
		children (aged 12 months to 13 years)	0.5×10^10^ CFU, twice a day for 12 weeks	lower SCORAD score at week 14 in the probiotic group than that in the placebo group; higher SCORAD score in the probiotic group from weeks 2 to 14 than that in the placebo group; *L. plantarum* CJLP133 significantly decreased total eosinophil count, logarithmic IFN-γ and IL-4	(Han et al., 2012)
	IS-10506	22 children with mild and moderate AD	10^10^ CFU, twice daily for 12 weeks	decreased SCORAD and levels of IL-4, IFN-γ, and IL-17; upregulation of Foxp3+ to IL-10 ratio	(Prakoeswa et al., 2017)
***L. sakei***	ProBio65	Children (aged 3–9 years) and adolescents (aged 10 to 18) with AD	1 × 10^10^ cells/d per sachet (400 mg)	decreased SCORAD total score when compared with baseline and potential improvement of skin barrier functions	(Rather et al., 2021)
	KCTC 10755BP	88 children (aged 2–10 years) with AEDS	5×10^9^ CFU, twice daily for 12 weeks	decreased total SCORAD scores	(Woo et al., 2010)
***L. reuteri***	ATCC55730	patients (aged 4–10 years)	10^8^ CFU/d, for 8 weeks were prescribed as 1 tablet once per day (2 hours before meals)	no significant differences	(Miniello et al., 2010)
		mothers and their babies	1×10^8^ CFU/d, from gestational week 36 until delivery	diminished IgE-associated eczema and skin prick test reactivity	(Abrahamsson et al., 2007)
***L. salivarius***	LS01	patients (aged 25–63 years)	5×10^9^ CFU/d, for a month	decreased SCORAD index and the count of Staphylococcus Aureus.	(Drago et al., 2014)
		43 patients (aged 0–11 years) with AD	1×10^9^ CFU/sachet, 2 sachets/d, for 8 weeks, and 1 sachet/day for the following 8 weeks	improved SCORAD score and itching	(Niccoli et al., 2014)
		38 patients (aged 18–46 years) with moderate/severe AD	1×10^9^ CFU/g, twice daily, for 16 weeks	decreased SCORAD score	(Drago et al., 2012)
		38 patients (aged from 18 to 46 years) with moderate/severe AD	1×10^9^ CFU/g, twice daily, for 16 weeks	improved SCORAD score and dermatology life quality index	(Drago et al., 2011)
	PM-A0006	60 children (aged 2–14 years) with moderate to severe AD	2×10^9^ CFU, twice daily, for 8 weeks	decreased SCORAD scores range 8 weeks and 10 weeks. *L. salivarius* PM-A0006 significantly reduced medication use frequency and eosinophil cationic protein levels at 8 weeks. *Lactobacillus salivarius* PM-A0006 reduced AD intensity	(Wu et al., 2012)
***L. paracasei***	CBAL74	58 infants and young children with moderate to severe AD (aged 6–36 months)	8 g/d for 12 weeks	decreased SCORAD index	(D'Auria et al., 2021)
	GM-080	infants with AD (aged 4–30 months)	1× 1,010 equivalent CFU, for 16 weeks	decreased CCL17 levels and TEWL in lesions and unaffected skin	(Yan et al., 2019)
	K71	34 adults with AD	100 mg/d (~2 × 10^11^ bacteria), over 12 weeks	decreased skin severity scores compared with baseline	(Moroi et al., 2011)
***L. casei***	DN-114001	40 children (aged 6–18 months) with AD	10^9^ cells/d, for 3 months	decreased SCORE index	(Klewicka et al., 2011)
***L. delbrueckii***	subsp. Bulgaricus LB-2	20 children (age 1–12 years) with AD	co-cultured with different concentrations of UV killed bacteria in RPMI-1640 plus 10% FCS for 48/72 h	upregulated the secretion of IL-10, IL-12, and IFN-γ, and decreased secretion of IL-4.	(Sheikhi et al., 2017)
***L. fermentum***	VRI 003 PCC™	53 children with moderate or severe AD	1×10^9^ twice daily for 8 weeks	increased Th1-type cytokine IFN-γ responses to PHA and SEB	(Prescott et al., 2005)

**Table 3:** Effects of *Lactobacillus* on the experimental AD.

Probiotic	strain	Experimental animal	Interventions	Outcome	Reference
***L. rhamnosus***	RHT3201	six-week-old female NC/Nga mice	1×10^8^, 1×10^9^, or 1×10^10^ cells/d, for 8 weeks.	improved dermatitis scores and frequency of scratching	(Lee et al., 2016)
	LGG	two litters of Beagles (same sire and dam) with AD	200 × 10^9^ CFU/d, ten Culturelle^®^ capsule; offspring from the second pregnancy, LGG, 100 × 10^9^ CFU/d	decreased allergen-specific IgE and partially prevented AD	(Marsella et al., 2012)
		2 adult beagles with severe AD and 16 pups	One capsule containing a minimum of LGG 20×10^9^ CFUs; first litter female dogs did not receive LGG. During the second pregnancy, a dosage of 10 capsules/d LGG from week 3 of gestation and continued throughout lactation. During the third pregnancy, 5 capsules/d from 3 weeks to 6 months of age	decreased serum titer of allergen-specific IgE and moderated reaction to intradermal testing	(Marsella, 2009)
		specific pathogen-free NC/Nga mice	30–50 mg/d	increased plasma IL-10 levels and enhanced IL-10 mRNA expression in both Peyer’s patches and mesenteric lymph nodes	(Sawada et al., 2007)
	CGMCC 1.3724 (LPR)	specific-pathogen free pregnant NC */* NgaTnd mice, pups until 12 weeks of age	5 ×10^8^ CFU*/* ml	decreased clinical symptoms of dermatitis, reduced scratching frequency	(Tanaka et al., 2009)
***L. acidophilus***	L-92	ICR mice BALB/c mice BALB/c and NC/Nga mice	3 and 30 mg/kg	inhibited vascular permeability in both passive cutaneous anaphylaxis	(Shah et al., 2010)
	L-55	female NC/Nga mice (5 weeks old) with AD-like skin lesions	1 and 10 mg/d, for 75 days	inhibited dermatitis score, ear swelling, scratching behavior	(Sunada et al., 2008)
***L. plantarum***	MG4221	NC/Nga mice (male, 4 weeks old)	a single dose (7 μg·cm^−2^) of 200 μL of PM2.5 (500 μg·mL^−1^) with 2% dinitrochlorobenzene another single dose (7 μg·cm^−2^) of 200 μL of PM2.5 (500 μg·mL^−1^) with 0.2% dinitrochlorobenzene	decreased transepidermal water loss and erythema; decreased scratching behavior	(Hong et al., 2021a)
	LM1004	AD-induced rat (histamine-induced vasodilation) and mouse (pruritus and contact dermatitis)	2 × 10^12^ cells, for 28 days	reduced vasodilation, pruritus, edema, and serum histamine	(Kim et al., 2019a)
	NCIMB8826	APOC1+/+ mice	3×10^8^ CFU, three times a week, for 8 weeks	ameliorated skin pathology, improved skin barrier integrity, eliminated of skin thickening, and fewer excoriations	(Mariman et al., 2016)
***L. sakei***	ProBio65	dogs with CAD	2 × 10^9^ CFU/g, for 2 months	reduced disease severity index, CASESI score values	(Kim et al., 2015a)
		25 male 6-week-old NC/Nga mice	5×10^9^ CFU/ml, 200 μL/d, for 2 weeks	improved condition of skin and reduced scratching frequency	(Kim et al., 2013)
		mice triggered by allergen	1 ×10^8^ CFU/mL, 200 μL/d, for 2 weeks	faster recovery of AD	(Park et al., 2008)
	WIKIM30	wild-type male BALB/c mice	2 × 10^9^ CFU bacteria, 200 μL/d	reduced AD-like skin lesions	(Kwon et al., 2018)
***L. reuteri***	Fn041	seven-week-old male and female BALB/C mice	1×10^9^ CFU/d, once a day, 100 μL each time, each time	suppressed AD symptoms such as skin swelling, mast cell and eosinophil infiltration	(Zhao et al., 2022)
	Japan Collection of Microorganisms 1112	specific pathogen-free male NC/Nga mice (aged 10 weeks)	0.1% (w/v) *Lactobacillus* water extract (LW)-treated, and 1.0% (w/v) LW-treated; 0.1% LW in 80% ethanol, or 1.0% LW in 80% ethanol, twice weekly for one week	suppressed the development of house dust mite-induced atopic skin lesions and thymus and activation-regulated chemokine expression	(Kawahara et al., 2018)
***L. paracasei***	KBL382	mice with Dermatophagoides farinae extract -induced AD	1 × 10^9^ CFU/d, for 4 weeks	reduced AD-associated skin lesions and epidermal thickening	(Kim et al., 2020a)
	K71	41 dogs with mild to moderate cAD	5 mg/kg, once daily, for 12 weeks	decreased CADESI, and pruritus scores; the reduced medication scores	(Ohshima-Terada et al., 2015)
	WK3001	five-week-old male NC/Nga mice	basic diet at concentrations of 0.03% (low dose) or 0.3% (high dose)	reduced development of AD-like skin lesions	(Wakabayashi et al., 2008)
***L. casei***	KCTC 12398BP	male NC/Nga mice	1, 10, and 100 μg/mL of P14 0.1, 0.2, 1, 5, and 10 μg/mL of P14	downregulated AD index and scratching score in AD-like NC/Nga mice	(Kim et al., 2015b)
	JCM 1134^T^	six-week-old male NC/Nga mice	NC/Nga mice were divided into four groups of six each and administered CD, DD (500 mg of dextran per day) LD (1×10^7^ CFU of lyophilized *L.* casei subsp. casei per day) LDD (1×10^7^ CFU of lyophilized *L.* casei subsp. casei and 500 mg of dextran per day) (control diet; CD) (dextran diet; DD) (*L.* casei subsp. casei diet; LD) (*L. casei* subsp. casei and dextran diet; LDD)	decreased clinical skin severity scores and total IgE levels	(Ogawa et al., 2006)
***L. delbrueckii***	OLL1073R-1	specific-pathogen-free female NC/Nga mice and BALB/c mice, (4- or 5-wk-old).	bacterial, 1 mg/d	inhibited development of dermatitis and elevation of an acute inflammation marker, serum amyloid A	(Kano et al., 2013)
	R-037	female BALB/c mice (5-weeks-old) and male NC/Nga mice (7-weeks-old)	5 g/d/mouse, from day 0 to day 55	reduced inflammatory auricular thickness and alleviated the AD clinical score	(Watanabe et al., 2009)
***L. johnsonii***	NCC533	NC/NgaTnd mice	4 weeks	suppressed exacerbation of the clinical severity of dermatitis and suppressed epidermal hyperplasia and infiltration of inflammatory cells in skin	(Tanaka et al., 2008)
		pups of 4 pregnant NC/Nga mice.	10^10^ cells, from 20 to 22 days of age *via* oral administration	enhanced gene expression of the proinflammatory cytokines [interleukin-8 (IL-8), IL-12 and IL-23] and decreased gene expression of CD86	(Inoue et al., 2007)
***L. brevis***	NS1401	female NC/Nga mice (24 Six-weeks old)	5 × 10^8^ CFU/d per mouse, for 8 weeks	reduced skin thickness and infiltration of mast cells and eosinophils in skin lesions and the size and number of immune cells in draining lymph nodes	(Choi et al., 2017)
	SBC8803	male 5-week-old NC/Nga mice	0%, 0.05% or 0.5%, once a week for 9 weeks.	inhibited ear swelling, and suppressed the development of dermatitis.	(Segawa et al., 2008a)

## 3 Monostrain *Lactobacillus* in the treatment and prevention of AD

### 3.1 *Lactobacillus rhamnosus* -effective prevention and treatment of AD in both animal and clinical experiments

*L. rhamnosus* is the most studied species of Lactobacillus (Petrova et al., 2021). The peptidoglycan of *L. rhamnosus* CRL1505 can regulate the immune function of human intestinal epithelial and dendritic cells (Salva et al., 2021). Early exposure to LGG in dogs with AD had long-term immune effects and significantly reduced allergen-specific IgE despite the lack of a clear clinical effect (Marsella, 2009; Marsella et al., 2012). In clinical trials, infants aged 0–2 years had a reduced incidence of atopic eczema when their mothers have been administered LGG during pregnancy (Kalliomäki et al., 2003). The preventive effect of LGG extended to 4 years (Kalliomäki et al., 2003). LGG may enhance the gut barrier function and promote immune response development in infants with AD (Nermes et al., 2011). In 2016, researchers observed that *L. rhamnosus* IDCC 3201 tyndallizate (RHT3201) had the potential to treat AD. The mast cell count and serum IgE concentration in axillary lymph node cells were decreased in RHT3201-fed NC/Nga mice compared with those in the control group (Lee et al., 2016). Another animal experiment concluded that heat-treated LGG could improve the symptoms of NC/Nga mice with AD (Sawada et al., 2007). In 2020, Jeong and colleagues (Jeong et al., 2020a) found that RHT3201 had a therapeutic effect on AD in children. *L. rhamnosus* GG (LGG) is a strain of *L. rhamnosus* that regulates gut flora and reduces conditional pathogenic bacteria (Chen et al., 2020b). When children with AD were supplemented with LGG, the clinical severity and quality of life improved (Carucci et al., 2022). Simultaneously, the use of topical steroids was reduced (Carucci et al., 2022).

*L. rhamnosus* decreases the concentrations of eosinophilic cationic protein, eosinophil count, IL-31 (Jeong et al., 2020a), and serum IgE (Tanaka et al., 2009; Lee et al., 2016); supports a better Th1/Th2 balance (Wickens et al., 2008); prevents pathogenesis caused by large molecules in the intestine; accelerates immune maturation (Nermes et al., 2011); stimulates peripheral blood cells to secrete IL-10 (Kalliomäki et al., 2003; Sawada et al., 2007); converts growth factor β (Kalliomäki et al., 2003), and upregulates the production of IFN-γ at the skin level (Tanaka et al., 2009). However, not all *L. rhamnosus* strains are equally effective. Wickens et al. (2012) studied the efficacy of *L. rhamnosus* HN001 and HN019 in atopic diseases and demonstrated that *L. rhamnosus* HN001 was more effective in improving AD than *L. rhamnosus* HN019 (Wickens et al., 2012). Moreover, the protective effect on eczema can persist two years when *L. rhamnosus* HN001 is administered to children with AD (Wickens et al., 2008). In addition, *L. rhamnosus* HN001 can prevent atopic sensitization in the long term (Wickens et al., 2013). Moreover, several studies have shown that the protective effect of LGG against AD requires further investigation. Two randomized controlled trials have shown that prenatal LGG treatment was not associated with a reduced risk of eczema (Boyle et al., 2008; Boyle et al., 2011). No clear causal relationship between the positive effects of LGG and infantile eczema has been reported (Fölster-Holst et al., 2006; Grüber et al., 2007; Kopp et al., 2008; Rose et al., 2010).

Some probiotics affect multiple immune pathways through different mechanisms and protect against the pathogenesis of eczema (Wickens et al., 2008). However, supplementation mothers with *L. rhamnosus* HN019, was not effective in preventing eczema in infants, indicating that *L. rhamnosus* HN019 is less likely to be passed on to infants through breast milk (indirect supplementation route) (Wickens et al., 2018). One of the reasons that prenatal LGG was not shown to prevent eczema in infants may be that the impact of prenatal probiotic therapy on fetal B cell development was not excluded (Boyle et al., 2008). Prenatal LGG intake may not directly contribute to the effects of postpartum breast milk regulation. It is possible that postpartum intake by nursing mothers can alter immunity and/or microbiota to benefit breast milk composition (Boyle et al., 2011). Probiotics are commonly added to dairy products (Grüber et al., 2007), which can also affect the efficacy of *L. rhamnosus* in AD. It is important to note that the number of participants who completed the study and the subgroups analyzed were insufficient. In addition, the complexity of human life makes it challenging to achieve homogeneity. In addition, eczema in early infancy can naturally improve, and more than 40% of patients with AD recover around the age of 3 years (Illi et al., 2004). Moreover, it is more difficult for clinical trials to recruit sufficient human participants than for animal studies to include a sufficient number of animals.

*L. rhamnosus* has been shown to be effective in the prevention and treatment of AD in both animal and clinical experiments. However, future research needs to continue to explore different strains of *L. rhamnosus* and use strains with excellent laboratory efficacy in clinical trials. Effective strains of *L. rhamnosus* can be passed on to the offspring from the mother, and subsequently, infants may potentially not develop AD.

### 3.2 Some strains of *Lactobacillus acidophilus* alleviate symptoms of AD and show good safety

*L. acidophilus* is a commercially significant probiotic isolated from the human gastrointestinal tract (Bull et al., 2013). Moreover, it is an important class of bioprotective agents (Anjum et al., 2014). Supplementation with *L. acidophilus* L-92 significantly reduced vascular permeability in diseased mice and attenuated the clinical symptoms of AD (Shah et al., 2010). The administration of *L. acidophilus* L-92 not only significantly attenuated AD symptoms in children (Torii et al., 2011), but also improved the symptoms in adults (Inoue et al., 2014). *L. acidophilus* triggers an anti-inflammatory response (Goh et al., 2021). *L. acidophilus* L-92 inhibited the inflammatory response dominated by Th2 cells by activating regulatory T (Treg) and Th1 cells (Yamamoto et al., 2016). *L. acidophilus* L-55 decreased the occurrence of anaphylactic dermatitis-like skin lesions in NC/Nga mice by decreasing serum total IgE levels (Sunada et al., 2008). However, the findings on several strains of *L. acidophilus* have been inconsistent. For example, there is no evidence that *L. acidophilus* NCFM can improve AD (Larsen et al., 2011). Supplementation with *L. acidophilus* LAVRI-A1 in children with allergies did not reduce the risk of dermatitis (Taylor et al., 2007; Prescott et al., 2008; Jensen et al., 2012), and the sensitization rate in the *L. acidophilus* LAVRI-A1 group was significantly higher than that in the control group (Taylor et al., 2007).

In early studies, *L. acidophilus* demonstrated good safety and efficacy in children with AD, and future studies in mothers are ongoing or are expected to begin soon. In addition, the time required to evaluate clinical outcomes is insufficient. The detailed interplay between the early microbial environment and the developing immune system remains largely unknown and requires further exploration. Overall, the findings suggest that not every strain of *L. acidophilus* has the potential to prevent or treat AD. The effect of *L. acidophilus* on the prevention of AD requires further study.

### 3.3 *Lactobacillus plantarum* regulates the host immune system to improve AD symptoms

*L. plantarum* is a rod-shaped lactic acid-producing bacterium that is used in probiotics and silage production. *L. plantarum* has the potential to be a highly effective immunomodulatory probiotic in the human gut microbiome. In recent years, an increasing number of studies have shown the health benefits of *L. plantarum*. (Seddik et al., 2017). The extract of fermented blueberry black rice containing *L. plantarum* MG4221 had an effect similar to that of dexamethasone, but with fewer side effects; oral administration of FBBBR in NC/Nga mice reduced skin dryness, erythema, and scratch behavior (Hong et al., 2021a). *L. plantarum* NCIMB8826 can soothe the skin of mice with AD, strengthen the skin barrier, and alleviate scratching (Mariman et al., 2016). Supplementation with *L. plantarum* CJLP133 and IS-10506 has been shown to be beneficial for treating AD in children (Han et al., 2012; Kim et al., 2017; Prakoeswa et al., 2017). In addition, *L. plantarum* IS-10506 has an immunomodulatory effect and can effectively relieve AD symptoms in adults (Prakoeswa et al., 2022).

*L. plantarum* BF_15 can successfully colonize murine intestines by rebalancing intestinal microbiota (Zhang et al., 2020). The extract of fermented blueberry black rice containing *L. plantarum* MG4221 inhibited the production of serum IgE and Th2 cell-related cytokines, suggesting that fermented blueberry black rice may be an essential functional food in AD (Hong et al., 2021a). Additionally, oral administration of *L. plantarum* NCIMB8826 reduced the number of mast cells in the colon. Finally, *Staphylococcus aureus* infection is the main reason for the exacerbation of AD-like symptoms, but *L. plantarum* can alleviate AD-like symptoms by inhibiting *Staphylococcus aureus* (Kim et al., 2020b). Additionally, lipoteichoic acids isolated from *L. plantarum* and *Staphylococcus aureus* have shown anti-AD effects. Lipoteichoic acid combination therapy can alleviate AD by reducing the formation of membrane attack complexes and inhibiting Th1 reactions (Kim et al., 2019c). Notably, *L. plantarum* LM1004 not only regulates the host immune system and gut microbiota, but is also promising for the treatment of AD and obesity in humans (Kim et al., 2019a). Collectively, the preliminary evidence suggests *L. plantarum* is a potential therapeutic strategy. Moreover, it has an acceptable safety profile in adults. *L. plantarum* improves the symptoms of AD, although it is ineffective in preventing AD. Future research should focus on the preventive effects of *L. plantarum* on AD. *L. plantarum* is a promising *Lactobacillus* strain that can be further explored to improve treatment options and efficacy.

### 3.4 *Lactobacillus sakei* has potential as a supplement for the treatment of AD due to its anti-inflammatory and skin barrier protective properties

*L. sakei* was isolated from fermented meat, fish, and kimchi. *L. sakei* KDP is a potent antioxidant and antibacterial agent (Ghoneum and Abdulmalek, 2021). *L. sakei* 07, combined with *Bifidobacterium bifidum* B10, regulates immunity and the gut microbiota (Wang et al., 2019). Oral administration of live and inactivate (Kim et al., 2013; Rather et al., 2021) *L. sakei* probio65 can increase skin sebum content and improve the function of the skin barrier (Rather et al., 2021). *L. sakei* probio65 inhibits AD-like skin lesions and may serve as an influential novel anti-inflammatory medication that resolves AD symptoms in mice (Kim et al., 2013). In experimental dogs (Kim et al., 2015a) and mice (Park et al., 2008) with AD, orally administered *L. sakei* probio65 significantly reduced the disease severity index without clear side effects. Supplemental treatment with *L. sakei* KCTC 10755BP has the potential to alleviate the clinical severity of AD syndrome in children (Woo et al., 2010).

*L. sakei* WIKIM30 was isolated from kimchi and can significantly reduce AD-like skin lesions, regulate allergic Th2 responses, increase the relative abundance of intestinal bacteria positively correlated with Treg production, and has potential in AD treatment (Kwon et al., 2018). Current research shows that *L. sakei* can be anti-inflammatory and can protect the skin barrier. *L. sakei* has the potential to be used as a therapeutic supplement in AD. *L. sakei* originates from fermented foods, and direct intake of fermented foods may have the same effect. As mentioned above, both live and inactivated *L. sakei* have been shown to be effective. Fermented foods such as kimchi are popular and readily available, and patients can effortlessly benefit and achieve improvement of AD. Future research can further apply *L. sakei* in clinical practice and investigate its preventive effect on AD. In addition, the therapeutic effects of other strains of *L. sakei* can also be explored.

### 3.5 *Lactobacillus reuteri* supplementation is effective in preventing AD

*L. reuteri* has been reported to occur naturally in the intestines of all vertebrates and mammals. *L. reuteri* induces neonatal IgA production (Mu et al., 2021). *L. reuteri* survives in the gastrointestinal tract of mammals and benefits host health (Engevik et al., 2021). *L. reuteri* NK33 can be used to improve gut dysbiosis (Han et al., 2020). *L. reuteri* strain, the Japan Collection of Microbiology 1112, significantly inhibited the expression of allergic lesions and thymus and activation-regulated chemokines at the site of lesions in NC/Nga mice (Kawahara et al., 2018). Prenatal and postnatal supplementation with *L. reuteri* Fn041 effectively prevented the fetus from developing AD, remodeled the intestinal ecology, and improved the immune function of Peyer’s patches (Qi et al., 2022; Zhou et al., 2022). *L. reuteri* Fn041 regulated the intestinal flora and significantly inhibited AD symptoms by regulating the systemic ratio of Th1 and Th2 cytokines in mice (Zhao et al., 2022). *L. reuteri* DYNDL22M62 attenuated AD symptoms by modulating gut bacteria in mice (Fang et al., 2022). Mothers and their babies supplied with *L. reuteri* ATCC 55730 had a lower prevalence of IgE-associated eczema at 2 years of age (Abrahamsson et al., 2007). However, in another clinical trial, *L. reuteri* ATCC 55730 did not improve clinical symptoms (Miniello et al., 2010). This may be because the small sample size. Alternatively, the duration of the experiment may have been too short. *L. reuteri* has been extracted from the gastrointestinal tract of all mammals, and future studies could explore the reasons for an absence of this *L.* and whether higher abundance of *L. reuteri* is beneficial. *L. reuteri* appears to have preventive and therapeutic effects in animals. Further clinical research on *L. reuteri* is required to explore the treatment and prevention of AD.

### 3.6 *Lactobacillus salivarius* actively improves the quality of life in children and adult patients with AD

*L. salivarius* is a Lactobacillus species that occurs in the human gastrointestinal tract and oral mucosa. It produces bacteriocins, and is used as a probiotic. It modifies the gastrointestinal system to alleviate intestinal diseases and promote host health (Neville and O'Toole, 2010). *L. salivarius* is valuable for both animals and humans. *L. salivarius* can reduce pathogen colonization of the gastrointestinal tract of animals (Hong et al., 2021b). Administration of *L. salivarius* can prevent and treat a variety of chronic diseases in humans (Chaves et al., 2017), including AD, asthma, cancer, and bad breath (Drago et al., 2014). The combined administration of *L. salivarius* PM-A0006 and fructooligosaccharides exhibited a notable anti-AD effect compared with either therapy alone in the treatment of children with moderate-to-severe AD (Wu et al., 2012). *L. salivarius* LS01 can help manage AD in children and improve their quality of life; moreover, partial effect remains after termination of medication (Niccoli et al., 2014). *L. salivarius* LS01 actively improves the quality of life in adult patients with AD by regulating the balance of Th1/Th2 (Drago et al., 2011; Drago et al., 2012; Kim et al., 2014). Additionally, *L. salivarius* has shown efficacy in improving AD in existing studies. As the name suggests, *L. salivarius* is present in the oral cavity. The study of the oral environment is of great significance for increasing *L. salivarius*. The preventive effect of *L. salivarius* on AD requires further study. However, the long-term safety and persistence of *L. salivarius* remains to be studied. More potent subspecies of *L. salivarius* are yet to be discovered.

### 3.7 *Lactobacillus paracasei* has anti-inflammatory properties that can reduce the development of AD-like skin lesions

*L. paracasei* originates from the healthy human gastrointestinal tract and is widely distributed in food. *L. paracasei* has anti-inflammatory properties that can reduce antigenic pro-inflammatory responses. *L. paracasei* improves immune function by enhancing NK cell function and IFN-γ concentrations (Lee et al., 2017). In addition to a preventive effect on AD-like skin changes in mice *L. paracasei* KW3110 demonstrated inhibitory effects even when supplementation was started after symptoms have appeared (Wakabayashi et al., 2008). Supplementation with *L. paracasei* KW3110 can significantly reduce the development of AD-like skin lesions in mice, while regulating immunity (Wakabayashi et al., 2008). Oral supplementation with *L. paracasei* K71 can be used to treat dogs with AD (Ohshima-Terada et al., 2015). A diet with added K71 can be used as a complementary therapy for adult AD patients (Moroi et al., 2011). Moreover, *L. paracasei* NL41 reduced inflammation by improving the intestinal environment and maintaining intestinal integrity in rats (Zeng et al., 2021). Daily oral administration of *L. plantarum* HEAL9 and *L. paracasei* 8700 has been shown to regulate the peripheral immune response in children with celiac disease autoimmunity (Håkansson et al., 2019). *L. paracasei* KBL382 can significantly reduce AD-related lesions and epidermal thickening in mice by modulating the immune response and changing intestinal microbiota composition(Kim et al., 2020a).

Additionally, heat-killed *L. paracasei* CBA L74 has a minimal effect on steroid use, but its effect on reducing the severity of AD needs to be further studied (D'Auria et al., 2021). However, there is no evidence that *L. paracasei* GM-080 has a retention effect equivalent to that of glucocorticoids (Yan et al., 2019). This may be due to the inappropriate selection of *L. paracasei* strains, timing of administration, timing of exposure, and failure to achieve appropriate dosing levels. In conclusion, *L. paracasei* enhances NK cell function and IFN-γ concentrations to regulate immune mechanisms and achieve anti-inflammatory effects. Concurrently, *L. paracasei* can improve the intestinal environment and maintain intestinal homeostasis to achieve anti-inflammatory effects. The duration of *L. paracasei* administration does not require special emphasis, and intervention in patients with AD before and after the appearance of symptoms can achieve the desired effect. Hence, these studies might help clarify whether *L. paracasei* can improve intestinal barrier function and maintain immune system balance. Oral administration of *L. paracasei* can prevent and treat AD. However, more clinical experiments are needed to explore prevention of AD with the administration of *L. paracasei*.

### 3.8 *Lactobacillus casei* treats AD by balancing the gut microbiota and immune responses

*L. casei* is found in many fermented foods and coexists with gut microbiota. It is involved in housekeeping functions, metabolism, cell wall biogenesis, and environmental adaptation (Licandro-Seraut et al., 2014). *L. casei* CCFM1074 can balance gut microbiota and immune responses (Fan et al., 2021). *L. casei* regulates the host immune response (Aktas et al., 2016) and can be used to treat AD. During the Japanese cedar pollen season, NC/Nga mice orally administered *L. casei* Japan Collection of Microorganisms (JCM) 1134T combined with dextran experienced a possible effect on the prevention and treatment of allergic reactions (Ogawa et al., 2006). Furthermore, researchers screened an active ingredient, protein P14, from the *L. casei* extract, which specifically lowered IgE and IL-4 levels in AD-like NC/Nga mice, suggesting potential therapeutic effects in AD (Kim et al., 2015b). Similarly, the administration of *L. casei* DN–114001 to the diet of children with AD was beneficial to the increase in the count of intestinal flora and its maintenance for five months after the cessation of probiotics (Klewicka et al., 2011). *L. casei* DN—114001 can improve clinical symptoms in children with AD long term (Klewicka et al., 2011). Overall, these studies provide a good foundation for developing future therapeutic or preventive approaches using *L. casei* in individuals with AD. *L. casei* has an extended effect after stopping supplementation; therefore, the effect of permanent colonization of the intestine after regular supplementation should be studied. Few studies have been conducted on *L. casei* for the treatment of AD. More subspecies of *L. casei* are yet to be identified.

### 3.9 *Lactobacillus delbrueckii* alleviates AD by maintaining and improving intestinal barrier function by stimulating immune cells to reduce inflammatory responses

*L. delbrueckii* is one of the most economically valuable fermented Lactobacillus species. *L. delbrueckii* maintains and improves the intestinal barrier function by stimulating immune cells (Kobayashi et al., 2019). *L. delbrueckii* also improved intestinal integrity and immune responses in piglets (Chen et al., 2020a). *L. delbrueckii* subsp. bulgaricus may be involved in regulation of immune factor secretion in patients with AD (Sheikhi et al., 2017). IL-6 is a leading cause of dermatitis; whereas oral administration with *L. delbrueckii* subspecies bulgaricus OLL1073R-1 attenuated dermatitis by inhibiting the IL-6 response and restoring the elevation of serum amyloid levels in the NC/Nga mouse model of AD (Kano et al., 2013). In addition, oral supplementation with heat-treated *L. delbrueckii* R-037 can inhibit the rise of serum total IgE in allergic model mice, decrease inflammation, and alleviate AD; however, its effect on serum total IgE levels needs to be further studied (Watanabe et al., 2009). Studies in animal (mouse) models and have shown that *L. delbrueckii* plays a role in the management of AD. Experimental samples are easier to obtain in animal experiments than in clinical studies. However, only results of clinical studies that show the efficacy of *L. delbrueckii* will enable its widespread use in the management of patients with AD. In future, it will be necessary to further investigate the use of *L. delbrueckii* in patients with AD, including factors such as the time of supplementation, dosage, and strain activity. Moreover, the understanding of the preventive function of *L. delbrueckii* requires further experiments in both animals and humans.

### 3.10 *Lactobacillus fermentum* regulates the immune response and benefits children with AD

*L. fermentum* is a gram-positive bacterium. It can improve the functionality and nutritional value of foods (Naghmouchi et al., 2020). *L. fermentum* can restore homeostasis of the intestinal microflora and regulate the immune response in mice (Rodríguez-Nogales et al., 2017). Mice were immunized with *L. fermentum* NWS29 and exposed to ovalbumin. *L. fermentum* NWS29 inhibited the expression of certain inflammatory factors to achieve an anti-inflammatory effect (Nawaz et al., 2015). Mice were inoculated with the Salmonella vaccine and *L. fermentum* PC2. When mice were challenged with live *Salmonella typhimurium*, *L. fermentum* enhanced mucosal and immune responses and played a protective role (Esvaran and Conway, 2012). *L. fermentum* KBL374 and KBL375 can modulate the innate immune response by improving intestinal barrier function and reducing leukocyte infiltration in mice (Jang et al., 2019). *L. fermentum* CJL-112 protected mice from the deadly influenza virus infection by stimulating macrophages, activating Th1 cells, and increasing immunoglobulin A production (Yeo et al., 2014). In a clinical trial, *L. fermentum* PCCTM strengthened Th1 IFN-γ responses and achieved clinical benefits in children with AD(Prescott et al., 2005).

*L. fermentum* MS15 inhibited exogenous IL-10 induced human β-defensin-2 and regulated the response to the inflammatory stimulus (Habil et al., 2014). In future, *L. fermentum* can be combined with other vaccines to enhance their protective effect (Esvaran and Conway, 2012). These different strains of *L. fermentum* have been shown to regulate immunity and may also have some effect in the prevention of AD. In future, more research is required to explore the potential of *L. fermentum* in the treatment of AD.

### 3.11 *Lactobacillus johnsonii* improves intestinal inflammation and alleviates the severity of AD*​*

*L. johnsonii* is a probiotic that can be isolated from dairy products. Notably, *L. johnsonii* BS15 can regulate intestinal inflammation (Charlet et al., 2020; Xin et al., 2020a; Xin et al., 2020b; Wang et al., 2021). Oral administration of *L. johnsonii* NC553 can relieve the severity of AD and inhibit epidermal hyperplasia and infiltration of inflammatory cells into the skin (Inoue et al., 2007). Additionally, it relieves skin damage by inhibiting pro-inflammatory cytokines and CD86 (Inoue et al., 2007). Therefore, early administration of *L. johnsonii* NC553 in mice with allergies may help reduce AD exacerbations (Tanaka et al., 2008). *L. johnsonii* has been shown to improve intestinal inflammation in animal models. *L. johnsonii* is present in fermented dairy products, and it is worth investigating whether an effective dose can be achieved through daily yogurt intake in children. *L. johnsonii* can also ameliorate skin damage in mice. However, there have been few experiments related to the treatment of AD with *L. johnsonii*, and further research is needed to determine its effectiveness in the prevention and treatment of AD.

### 3.12 *Lactobacillus pentosus* regulates the host immune system and improves systemic inflammatory response

*L. pentosus* regulates the host immune system and plays an integral role in intestinal health (Ma et al., 2020). *L. pentosus* KF340 regulates systemic immunity and improves systemic inflammatory response (Kim et al., 2019b). *L. pentosus* S-PT84 may be involved in the modulation of immune mechanisms to alleviate clinical allergy symptoms (Majumder et al., 2020). Although administration of *L. pentosus* and placebo can improve symptoms, *L. pentosus* significantly improved the average subjective ratings evaluated using the SCORAD index for allergen-sensitizing AD (Ahn et al., 2020). *L. pentosus* KF340 reduced cell infiltration and serum IgE levels at the site of lesions in mice by inducing type 1 regulatory T cells (Tr1 cells) that produce IL-10 (Kim et al., 2019b). *L. pentosus* S-PT84 reduced the concentrations of histamine in the serum, mouse mast cell protease, total IgE, and IgG (Majumder et al., 2020). The detailed function of *L. pentosus* remains largely unknown and requires further investigation. *L. pentosus* has been shown to exert anti-inflammatory effects and benefit intestinal health. However, there is limited evidence on the efficacy of *L. pentosus* in the treatment and prevention of AD, and further research is needed.

### 3.13 *Lactobacillus brevis* alleviates symptoms of AD by regulating the immune response*​*

*L. brevis* is a gram-positive, rod-shaped *Lactobacillus* that is frequently used as a starter culture in silage fermentation, sourdough, and lactic acid-fermented beer and wine. *L. brevis* is widely used in the fermentation industry. Orally administered *L. brevis* SBC8803 can significantly inhibit the production of IgE and severity of AD symptoms, and long-term use can inhibit AD development. However, it did not affect the production of cytokines produced by Th1 and Th2 (Segawa et al., 2008a). *L. brevis* NS1401, isolated from kimchi, stimulated immune cells to secrete Th1 or Th2 cytokines, balance Th1/Th2, and alleviate AD symptoms (Choi et al., 2017). *L. brevis* stabilizes the gut microbiota, prevents the growth of pathogenic bacteria, and reduces intestinal inflammation (Han et al., 2021). *L. brevis* can not only improve disease but also regulate immunity. In nine-week-old female BALB/c mice managed with *L. brevis* KB290 (3 × 109 CFU/g), cytotoxicity mediated by mouse splenocytes increased (Fukui et al., 2013). Similarly, the spleen cells of mice treated with *L. brevis* KCTC12777BP also expressed high levels of TNF-α. *L. brevis* 12777BP improves immunity in mice and prevents organisms from being invaded by pathogens (Jeong et al., 2020b). The supernatant of *L. brevis* BGZLS10-17 can be divided into two components: a GABA-containing and a GABA-free component (Bajić et al., 2020). The supernatant containing GABA relies on ATG5 autophagy to stimulate Foxp3+, IL-10, and transforming growth factor-β, CTLA4 and Sirp-α isoimmunoregulatory molecule expression (Bajić et al., 2020). The GABA-free supernatant can also regulate the immune response through other mechanisms (Bajić et al., 2020). Heated *L. brevis* KB290 accelerated the secretion of IL-8, induced ERK1/2 phosphorylation, increase p38MAPK phosphorylation, and enhanced the expression of IL-8 mRNA (Yakabe et al., 2013). Heated *L. fermentans* SBC8803 inhibited IgE production and histamine secretion (Segawa et al., 2008b). *L. brevis* regulates immunity through various mechanisms and has anti-inflammatory effects. Unfortunately, the prevention and treatment of AD by *L. brevis* remain unclear. *L. brevis* may be an innate probiotic to inhibit AD development, and is a promising *Lactobacillus* strain that requires further research.

## 4 Multi-strain *Lactobacillus* for the treatment and prevention of AD

By critically reviewing the current literature, we also address the advantages and major disadvantages of the simultaneous use of two or more strains of probiotics. *Lactobacillus* supplementation can improve AD by regulating the intestinal microbiome. Intestinal flora has the potential to improve AD. In two experiments, administration of a *Lactobacillus* mixture had a preventive effect on AD in hairless mice (Holowacz et al., 2018a; Holowacz et al., 2018b). *L. plantarum* CJLP55, CJLP133, and CJLP136 isolated from kimchi inhibited AD-like skin lesions, reduced serum IgE levels, and restored the condition of the skin (Won et al., 2011). Moreover, multi-strain probiotics have been shown to have immunomodulatory effects and prevent AD in high-risk infants (Kukkonen et al., 2007). A mixture of heat-inactivated *L. casei* LOCK 0900, *L. casei* LOCK 0908, and *L. paracasei* LOCK 0919 modulated *in vitro* cytokine profiles of allergic children to produce anti-allergic Th1 reactions (Cukrowska et al., 2010). More specifically, prenatal and postnatal supplementation with a mixture of *Bifidobacterium* BGN4, *L.* aD011, and *Acidophilus* A031 can substantially reduce the probability of developing AD before the age of one year (Kim et al., 2010). Compared to a single strain, mixed lactic acid bacteria significantly enhanced the ability of Th1 cells to respond. Multi-strain probiotics help maintain good skin function, are beneficial for the treatment of AD (Rosenfeldt et al., 2003; Wang and Wang, 2015), and maintain intestinal barrier function in children with AD (Rosenfeldt et al., 2004). In addition, a mixture of *Lactobacillus* spp. improves the clinical symptoms of adults with AD (Iemoli et al., 2012).

However, benefits of the use of mixed probiotics should be interpreted with caution. Consumption of formula containing probiotics (*Bifidobacterium longum* BL999 and *L. rhamnosus* LPR) before the age of one year did not effectively prevent eczema in infants at high risk of allergies in Asia (Soh et al., 2009). Eczema symptoms in infants did not change when *L. paracasei* CNCM I-2116 or *Bifidobacterium lactate* CNCM I3446 were used as an adjunct to basic topical therapy (Gore et al., 2012). Moreover, there is no evidence that *L.* NFCM and *L.* Bi-07 affect the intestinal flora of children with AD (Larsen et al., 2011). The combination of probiotics did not have a positive effect on AD treatment, which may be due to several reasons. First, there were differences between strains, different combinations of strains, and individual differences in the study subjects. Second, although the researchers prudently selected the infants, irreversible immune-related events occurred. Therefore, probiotic supplementation had no effect on AD. Moreover, of the reason for the probiotic mixture not achieving the desired effect may be because the mother did not supplement with probiotics before antenatal administration. Asian populations may differ, as this is the first randomized controlled trial to be conducted in Asia (Soh et al., 2009). In addition, the lack of effect may be due to a smaller bacterial population and a smaller number of experimental subjects (Larsen et al., 2011). *Lactobacillus* species are complex, the strains are diverse, and their combinations are random and varied. Countless combinations of different strains of the same or different species occur. Perhaps, we can find an effective way to explore the therapeutic effects of these different combinations of strains on AD. Inappropriate strains can have negative effects. Perhaps strains with side effects negate the efficacy of beneficial strains, which needs to be explored further. Moreover, research in Asian populations is limited. The efficacy of mixed strains in AD treatment and prevention should be investigated in Asia, a region with a large population.

## 5 Conclusion

Based on reviews and meta-analyses, probiotics can prevent or treat AD, and perinatal administration of probiotics can prevent AD (Kuitunen, 2013; Mansfield et al., 2014; Panduru et al., 2015). Children (Tan-Lim et al., 2021) and adults with moderate to severe AD can also be administered probiotics to treat AD (Kim et al., 2014). In addition, the preventive effect of probiotics on pediatric AD is better than that of treatment (Lee et al., 2008). A mixture of *Lactobacillus* and *Bifidobacteria* can effectively reduce the incidence of eczema in infants and young children during the first three years of life (Sun et al., 2021). The preventive effects of probiotics on eczema appear to last until age of two (Dang et al., 2013) and extend to the age four years (Kuitunen, 2013). The variety and specificity of probiotic strains enriches therapeutic options. Nevertheless, there is substantial evidence that LGG supplementation does not reduce the prevalence of eczema (Kim et al., 2014). Not all *Lactobacillus* strains can be used to treat AD, and further experiments are needed to screen for the most effective *Lactobacillus* strains. Additionally, probiotics require repeated experiments to determine the mechanism of their efficacy against AD, effective dose, optimal administration time, and other characteristics.

In conclusion, since the discovery of *Lactobacillus*, numerous studies have demonstrated that this bacterium has a positive effect on the host (**Tables 2**, **3**). In addition, previous research demonstrated the robust anti-inflammatory and homeostatic effects of *Lactobacillus*. The various health properties of *Lactobacillus* have been confirmed by different research groups. The mechanisms and beneficial effects of *Lactobacillus* are diverse (e.g., inflammation, immunity, gut health, brain function). Importantly, various types, species, and strains of *Lactobacillus exist*. Different strains from the same species have different functions. Therefore, research findings must be considered with utmost caution. The effect of some *Lactobacillius*, such as *L. rhamnosus* HN001 and *L. casei*, is retained for longer after supplementation; therefore, extending their function and permanent colonization of the intestine after regular supplementation can be studied. A portion of Lactobacillus is isolated from human organs. The absence of normal *Lactobacillus* colonization in some patients and options for restoration of these *Lactobacillus* strains are worth examining. In addition, different subtypes of the same *Lactobacillus* strain may have opposite effects; therefore, the positive and negative effects of the subtypes remain to be studied. A more potent subtype may suppress the effects of the effective subtype. However, most of the *Lactobacillus* strains are commensal or are present in food. Thus, ruling out an external interference in experiments is challenging. Finally, as a promising step towards precision and personalized medicine, *Lactobacillus* may become a food supplement to improve future AD treatments. Additional research is needed to explore other varieties of probiotic strains to enrich treatment options. Moreover, effective methods to preserve the activity and effects of probiotics should be explored. Importantly, additional human studies are needed to support the growing evidence of the beneficial effects observed in animal models of various diseases such as cancer, depression, and obesity.

## Author contributions

AX is responsible for the collection of data and writing of the original manuscript. AC and YC are responsible for the organization of the original manuscript. ZL and SJ are responsible for editing. DC and RY are responsible for the concept development, review of the manuscript and revision. RY is responsible for funding acquisition. All authors contributed to the article and approved the submitted version. 
